# From the Annual Report of the Secretary of Agriculture to the Governor for the Year Ending December 31, 1902

**Published:** 1903-01

**Authors:** 


### SANITARY CONTROL WORK.

### FROM THE ANNUAL REPORT OF THE SECRETARY OF AGRICULTURE TO THE GOVERNOR FOR THE YEAR ENDING DECEMBER 31, 1902.

The office of the State Veterinarian has experienced a year of unusual activity and progress. As heretofore, the greater part of the State Veterinarian's work has consisted in aiding herd owners who wish to eradicate tuberculosis from their herds and, with this object in view, enter voluntarily into a contract with this Commonwealth under which they receive important assistance on condition that they will do what they can to make the work permanent. Other diseases of animals have also required much attention during the year. "Rinderseuche" has been among the most important, because the most prevalent. There has been less anthrax and black-quarter than for several years, due, it is believed, to the systematic vaccination against these diseases that has been practised on rather a large scale during the past few years.

Ever since the organization of the work of the State Live-stock Sanitary Board a laboratory has been maintained for the production of the tuberculin, mallein, and anthrax vaccine used in the regular work of the board. The cost of maintaining the laboratory, has been more than made up in the value of these products. But in addition to this work, the laboratory has served a most useful purpose in affording facilities for the diagnosis of specimens from diseased animals, and has by this service considerably increased the efficiency of the veterinary profession of the State. Outside of all this routine work the laboratory has furnished the opportunity for conducting a rather remarkable volume of research. This division of the work has been most fruitful, and has been the means of revealing many new and serviceable facts in connection with the pathology, the diagnosis, and the prevention of some of our most prevalent and destructive animal diseases. The most notable achievements in this line during the past year have been, first, the establishing of the essential identity of the animal and human tuberculosis; second, the development of a method for immunizing cattle against tuberculosis by vaccination, and, third, the identification of the "Rinderseuche" as the disease that has heretofore been called the "mountain disease of cattle," and has caused considerable losses in some of the rougher parts of the State.

In regard to tuberculosis, the number of inspections is limited only by the funds available for making them. There are several times as many applications from herd owners as can be responded to under the existing financial limitations. Since all herds whose owners apply for an inspection and tuberculin test cannot be inspected and tested, and all tubercular cattle removed as is desired, the attempt is at all times made to test the herds that appear to be most extensively infected, and in respect to other herds, to remove the animals that are most immediately dangerous. In this way 912 tubercular cattle have been condemned and destroyed during the year. These were appraised at $26,306.25. There is at present a most active desire on the part of cattle owners to free their herds from tuberculosis. They appear to keenly realize the harm the disease is doing, and are willing to co-operate with the State Veterinarian to repress it. Indeed, as stated above, they constantly apply, of their own volition, for more aid than the State Live-stock Sanitary Board can pay for. The result of this interest is that tuberculosis of cattle is being repressed in Pennsylvania at a gratifying rate, and each year shows less loss from this cause.

There have been thirty-five cases of glanders in the State during the past year. Most of these diseased animals were involved in two outbreaks of the disease, one of which came from a glandered mule from East St. Louis, and the other was traced to a pony from Maryland. By immediately checking such outbreaks, the dissemination of the disease and the infection of the horse stock of the State is prevented. The value of this protection is apparent when it is known that in Massachusetts, with about one-fifth the area of

Pennsylvania, there are from seven to eight hundred condemnations on account of glanders each year.

Rabies has prevailed rather extensively, and nearly all parts of the State have been involved. It is extremely difficult to prevent this disease, and it is scarcely possible without establishing a general quarantine of all dogs in infected districts. In order that this may be done effectually additional legislation is necessary, and a bill covering this ground will be submitted to the incoming Legislature.

The discovery of "Rinderseuch" has cleared up a question as to the identity of a disease that has prevailed rather extensively among cattle turned out in some of the rougher mountainous parts of the State. This disease has been shown to exist in only a few other parts of the United States, but now that attention has been called to it, it may be found that it is of much wider distribution than has been supposed.

All things considered, the most notable achievement of the year, so far as the work of the State Veterinarian is concerned, is the development of a means to vaccinate cattle for the prevention of tuberculosis. This process consists in introducing into the system of the animal a culture of the tubercle bacillus so attenuated as to be incapable of producing disease. After a few treatments of this sort the animal is able to resist, without a sign of the least injury, inoculation with virulent tubercular material that is fatal for unprotected animals. This work has gone far enough to show that the principle upon which it is based is sound. It now remains to develop the methods that are necessary to make this system of general service. The State Veterinarian and his assistant, Dr. S. H. Gilliland, are now actively at work on this problem and are of the belief that they will soon be able to solve it. The immense practical value of this discovery cannot now be fully estimated. It is clear, however, that successful vaccination against tuberculosis of cattle will save the State annually far more than the entire cost of the work of the State Live-stock Sanitary Board and of the entire Department of Agriculture. The State is to be congratulated upon the foresight of the Legislature that led to the maintenance of the laboratory and research work of the State Live-stock Sanitary Board. It has already paid for itself many fold in laboratory products for use in diagnosticating and preventing disease and in other actual results now to its credit.

---

Dr. W. Horace Hoskins, of Philadelphia, Pa., made a tour of Columbia County, Pennsylvania, for the State Live-stock Sanitary Board, in November, investigating an epizoötic outbreak of hog cholera.

